# Vancouver call for action to strengthen expertise in radiological protection worldwide

**DOI:** 10.1007/s00411-023-01024-5

**Published:** 2023-04-25

**Authors:** W. Rühm, K. Cho, C.-M. Larsson, A. Wojcik, C. Clement, K. Applegate, F. Bochud, S. Bouffler, D. Cool, G. Hirth, M. Kai, D. Laurier, S. Liu, S. Romanov, T. Schneider

**Affiliations:** 1 Helmholtz Centre Munich, German Research Centre for Environmental Health, Ingolstädter Landstraße 1, 85764 Neuherberg, Germany; 2 Korea Institute of Nuclear Safety, Yuseong, 114, Daejeon, 34142 Korea; 3 Australian Radiation Protection and Nuclear Safety Agency, 619 Lower Plenty Road, Yallambie, VIC 3085 Australia; 4 Centre for Radiation Protection Research, Stockholm University, Svante Arrheniusväg 20C, 106 91 Stockholm, Sweden; 5 Institute of Biology, Jan Kochanoski University, 25-406 Kielce, Poland; 6 International Commission on Radiological Protection, 280 Slater Street, Ottawa, ON K1P 5S9 Canada; 7 University of Kentucky College Medicine, 800 Rose Street MN 150, Lexington, KY 40506 USA; 8 Institute of Radiation Physics, Lausanne University Hospital and University of Lausanne, Rue du Grand-Pré 1, 1007 Lausanne, Switzerland; 9 Radiation Protection Sciences Division, UK Health Security Agency, Didcot, OX11 0RQ Oxon UK; 10 Nippon Bunri University, 1727 Ichigi, Ōita, 870-0397 Japan; 11 Institut de Radioprotection et de Sûreté Nucléaire, BP 17-92262 Fontenay-aux-Roses Cedex, 31 Avenue de la Division Leclerc , 92260 Fontenay-aux-Roses, Île-de-France France; 12 China Institute of Atomic Energy, 275 (1), Beijing, 102413 People’s Republic of China; 13 Southern Urals Biophysics Institute, Ozyorsk, Chelyabinsk Region Russian Federation; 14 Nuclear Protection Evaluation Centre, 28, rue de la Redoute, 92260 Fontenay aux Roses, France

**Keywords:** Radiological protection, Radiation research, Education and training, Competence

## Abstract

Ionising radiation has been used for over a century for peaceful purposes, revolutionising health care and promoting well-being through its application in industry, science, and medicine. For almost as long, the International Commission on Radiological Protection (ICRP) has promoted understanding of health and environmental risks of ionising radiation and developed a protection system that enables the safe use of ionising radiation in justified and beneficial practices, providing protection from all sources of radiation. However, we are concerned that a shortage of investment in training, education, research, and infrastructure seen in many sectors and countries may compromise society’s ability to properly manage radiation risks, leading to unjustified exposure to or unwarranted fear of radiation, impacting the physical, mental, and social well-being of our peoples. This could unduly limit the potential for research and development in new radiation technologies (healthcare, energy, and the environment) for beneficial purposes. ICRP therefore calls for action to strengthen expertise in radiological protection worldwide through: (1) National governments and funding agencies strengthening resources for radiological protection research allocated by governments and international organisations, (2) National research laboratories and other institutions launching and sustaining long-term research programmes, (3) Universities developing undergraduate and graduate university programmes and making students aware of job opportunities in radiation-related fields, (4) Using plain language when interacting with the public and decision makers about radiological protection, and (5) Fostering general awareness of proper uses of radiation and radiological protection through education and training of information multipliers. The draft call was discussed with international organisations in formal relations with ICRP in October 2022 at the European Radiation Protection Week in Estoril, Portugal, and the final call announced at the 6th International Symposium on the System of Radiological Protection of ICRP in November 2022 in Vancouver, Canada.

## Vancouver call for action

At its symposium in Vancouver in 2022, ICRP called for action to strengthen expertise in radiological protection worldwide through:

- National governments and funding agencies strengthening resources for radiological protection research allocated by governments and international organisations,
- National research laboratories and other institutions launching and sustaining long-term research programmes,
- Universities developing undergraduate and graduate university programmes and making students aware of job opportunities in radiation-related fields,
- Using plain language when interacting with the public and decision makers about radiological protection, and,
- Fostering general awareness of radiation and radiological protection through education and training of information multipliers.

## International commission on radiological protection

The International Commission on Radiological Protection (ICRP) is a charity registered (#1166304) with the Charity Commission for England and Wales with the objective to “advance for the public benefit the science of radiological protection, by providing recommendations and guidance on all aspects of protection against ionising radiation, without unduly limiting beneficial practices that give rise to exposure to radiation.”

Since its foundation in 1928, ICRP has developed—as an independent, not-for-profit, and non-governmental organisation—the System of Radiological Protection (the “System”), used globally in all situations where exposure to ionising radiation occurs. This System has supported development and implementation of justified and beneficial technologies that use ionising radiation, while ensuring protection of people and the environment against detrimental consequences of their use. It has also supported protection from natural sources of ionising radiation. ICRP is neutral regarding national policies and operates independently of any promoting interests. The System, based on the most recent scientific evidence as reviewed by the United Nations Scientific Committee on the Effects of Atomic Radiation (UNSCEAR), has been the basis for international safety standards developed by the International Atomic Energy Agency (IAEA) and other organisations, and integrated in national legislation all over the world.

## The situation

### Threat of missed opportunities

While the System of Radiological Protection is robust and globally recognised, its implementation is dependent upon knowledgeable and engaged people. ICRP is increasingly concerned about the infrastructure to provide well qualified individuals to the radiological protection community in several countries. For example, in the 2012 Bonn Call-for-Action, the International Atomic Energy Agency (IAEA) and the World Health Organisation (WHO) stressed that for the strengthening of radiological protection in medicine there is “a need for a holistic approach which includes partnership of national governments, civil society, international agencies, researchers, educators, institutions and professional associations aiming at identifying, advocating and implementing solutions to address existing and emerging challenges; and leadership, harmonisation and co-ordination of activities and procedures at an international level” (IAEA-WHO 2012). In the United States, the National Council on Radiation Protection and Measurement stated that the “looming shortage of radiation professionals represents a serious threat to the United States: scientific leadership is being lost, competition in world markets is affected, and protection of our citizens and country diminished.” (NCRP 2015). An international consortium that reviewed the funding for radiation research in several countries concluded that a “better understanding of the biological consequences of radiation exposure is becoming more important with increasing public concerns on radiation risks and other radiation literacy. Continued funding for radiation research is needed, and education and training in this field are also important” (Cho et al. 2019). European Union researchers warned that many states “have lost key competences and are no longer capable of independently retaining their current research activities in radiation sciences, with implications for effectively fulfilling operational and policy needs and obligations” (Ottolenghi et al. 2019) so investment in education and training is essential (Salomaa et al. 2017). Recently, the German Radiation Protection Commission has expressed their view that “German research would greatly benefit if scientific competence in those research areas identified to be important for radiation science be rebuilt, kept, and strengthened (translation from German)” (SSK 2021). Both the IAEA and the US National Academy of Sciences have called for radiation science and protection education for all undergraduates in health sciences (Vassileva et al. 2021; Linet et al. 2022). Very recently, the US National Academy of Sciences evaluated the status and needs for increased resources for restarting the federal low-dose radiation research program in the United States (NAS 2022).

These concerns reflect developments that continue to take place while the applicability of radiation/nuclear technologies is growing, for example in medicine, and there is a strong desire among developing countries to access such technologies for the benefit of their people. The resource limitations seen in many sectors and countries may be related to a variety of factors, including national policies regarding electricity generation from nuclear power, a mistaken perception that everything is under control and no work is needed, or due to the underestimation or lack of knowledge of the role that the beneficial use of ionising radiation plays in many sectors of science and society today. Therefore, if not mitigated, there is a risk for reduced interest of students in radiation sciences and a growing shortage of expertise in radiological protection that can be drawn upon for managing radiation risks associated with both current and future technologies and practices.

### Relevance of radiological protection for the UN sustainable development goals

Radiological protection is a highly multidisciplinary undertaking, so the concerns raised result in a wide variety of potential impacts covered by several UN Sustainable Development Goals (SDGs). For example, if continued, any trend of decreasing expertise in radiological protection and safety may have a direct, negative impact on the UN Sustainable Development Goal (SDG) #3 “Good Health and Well-being”, ensuring healthy lives and promoting well-being for people at all ages, keeping in mind the central role ionising radiation plays in many medical applications covering imaging and cancer therapy. Obviously, SDG #4 “Quality Education” is central to this issue. Promoting inclusive and equitable quality education is an important factor addressing the decreasing global capacity for research and development of professional expertise in radiological protection. In addition, there is a significant risk of exacerbating the existing inequalities in radiological protection capacities in various parts of the world, setting back the SDG #10 “Reduced Inequalities” within and among countries. The System of Radiological Protection addresses protection not only of humans but also of non-human biota in the environment from detrimental effects of exposure to radiation. Consequently, expertise in radiological protection directly relates to SDG #14 Life Below Water and #15 Life on Land.

### Opportunities for a modern society

For several societal sectors, the implementation of safe radiation technologies is of major importance, in particular for the health sector. As science and technology move forward, new applications involving ionising radiation such as innovative medical diagnostic and treatment modalities, development of new materials, new radiation detection technologies, advanced long-term space exploration missions, long-term safety of disposal of radioactive waste, and many more are continuously developed. To make such developments successful, experts are required who have an appropriate scientific and practical background, and an understanding of the practical implementation and application of the system of protection and the procedures that enhance radiological protection.

However, not only new technologies result in exposure to ionising radiation. For example, radon (a natural radioactive noble gas) is present in dwellings and workplaces. Because it is the second leading cause of lung cancer after smoking, radon poses a major health problem for the public in many countries of the world, as well as in occupational settings. Many activities, including mining and extraction of oil and gas, generate by-products with enhanced levels of natural radioactive materials which require special consideration to avoid harm to people and the environment. Furthermore, natural radioactive material in the food chain contributes to internal radiation exposures of people and non-human species, while natural cosmic radiation leads to increased radiation doses particularly for aircrew and aircraft passengers.

Most of these naturally occurring radiation exposures are considered low dose, and the incidence of health effects induced by such radiation doses is so low as to be unobservable. This contrasts with the anxiety that the word “radiation” can trigger. However, because sufficient epidemiological evidence in this dose region is lacking, or is inconclusive, large uncertainties prevail over the shape of the dose response curve in the low and very low-dose region for radiogenic cancer, with opinions spanning from the existence of a threshold below which there is no excess cancer risk to the belief that, per unit dose, low doses are more detrimental than high doses. The disagreement among scientists is a driver of progress in radiation research, like in any field of science. If not properly understood as such, however, it could be interpreted as weakness, creating anxiety, and opening the door for those who profess to offer overly simplistic solutions to complex problems. The consequence could be, on the one hand, reduced safety of radiation technologies or, on the other hand, their rejection. Either consequence can have negative effects for citizens and society. Hence, it is particularly important to ensure that a sufficiently high level of competence exists that builds on thorough quality assurance and metrological traceability and that can serve as a trustworthy source of information for stakeholders and the public. It is well known that a high level of public trust is the basis of social capital—an essential element of a well-functioning society. For the proper uses of ionising radiation, the easy access to credible information empowers people to make their own informed choices, based on understanding and respect for the potential benefits and risks, to reduce anxiety while keeping vigilance. It is important to bear in mind that earning public trust is a long process and that it is quickly lost if both competence and two-way communication strategies are not maintained (Covello 2011).

### ICRP actions to promote radiological protection expertise

ICRP promotes radiological protection through several activities. For example, ICRP prepares publications including general recommendations on the System of Radiological Protection and guidance in planned, emergency, and existing exposure situations. Since 2020, these publications have been made freely available to everyone in the world two years after each is released. ICRP publications are complemented by biennial symposia, published research priorities to be considered by the international scientific community, and dissemination of easy-to-read information through ICRPædia (www.icrpaedia.org). In 2019, ICRP established a mentorship programme to engage university students and early-career professionals and scientists as mentees in ICRP Task Groups with the guidance of an ICRP member as mentor.

Dissemination of information is now further enhanced through open webinars and digital workshops where key elements of the System as well as current activities of ICRP are shared. Plans to refine the System have recently been presented and openly discussed at a Workshop, to engage in the review and revision of the System. At the Workshop, the need for education and training in radiation research and radiological protection was highlighted by many participants (Rühm et al. 2022).

While these and similar activities of ICRP will be continued and expanded, being a charity with limited financial resources, ICRP emphasises that much greater national and international efforts and collaborations are indispensable to strengthen research, infrastructure, education and training in radiation sciences and radiological protection worldwide, requiring joint actions by governmental and non-governmental organisations. We emphasise that research and training go hand in hand; sufficient financial resources for radiation research are essential for successful academic and professional training programmes.

## Vancouver call for action to strengthen expertise in radiological protection worldwide

Motivated by the reasons summarised above, ICRP has recently prepared a draft paper highlighting the importance to strengthen expertise in radiological protection worldwide. This draft paper was discussed at a meeting with international organisations in formal relations with ICRP in September 2022, which had been organised in conjunction with the European Radiation Protection Week in Estoril, Portugal. Organisations who contributed to that meeting included Conference of Radiation Control Program Director (CRCPD), European ALARA Network (EAN), European Alliance for Medical Radiation Protection Research (EURAMED), European Association of National Metrology Institutes (EURAMET), European Association of Nuclear Medicine (EANM), European Federation of Organisations for Medical Physics (EFOMP), European Nuclear Installations Safety Standards Initiative (ENISS), European Platform on Preparedness for Nuclear and Radiological Emergency (NERIS), European Radiation Dosimetry Group (EURADOS), European Radioecology Alliance (ALLIANCE), European Society of Radiology (ESR), Heads of the European Radiological Protection Competent Authorities (HERCA), Ibero American Forum of Radiological and Nuclear Regulatory Organisations (FORO), IEC Electrical Nuclear Instrumentation (IEC/TC45), International Atomic Energy Agency (IAEA), International Commission on Radiation Units and Measurements (ICRU), International Radiation Protection Association (IRPA), International Society of Radiology (ISR), Multidisciplinary European Low Dose Initiative (MELODI), National Council on Radiation Protection and Measurements (NCRP), OECD Nuclear Energy Agency (NEA), World Nuclear Association (WNA).

Feedback obtained at that meeting was taken into account and the call for action was announced at ICRP’s 6th International Symposium on the System of Radiological Protection on 7th November 2022 in Vancouver, Canada. Specifically, ICRP called for action to strengthen expertise in radiological protection worldwide through:

### National governments and funding agencies strengthening resources for radiological protection research allocated by governments and international organisations

Enhancement of radiation sciences should be a long-term societal goal with involvement and benefit across numerous scientific disciplines. This will facilitate the use of ionising radiation in various areas where benefits to society will outweigh any radiation-related risks, support development of new technologies involving ionising radiation, improve health and well-being, and support strengthening of societal resilience, for example through improved emergency preparedness for multi-factorial crises.

### National research laboratories and other institutions launching and sustaining long-term research programmes

For example, national research laboratories should include topics relevant for radiological protection, taking advantage of new and modern technological developments that result in reduced exposures to medical patients, workers, and the public. This would also strengthen knowledge of radiation risks and contribute to an improved scientific foundation of the recommendations issued by ICRP. Such efforts could build on the areas of research identified by ICRP to strengthen the System of Radiological Protection (Laurier et al. 2021).

### Universities developing undergraduate and graduate university programmes and making students aware of job opportunities in radiation-related fields

These could include attractive bachelor, master, PhD and post-doctoral projects in natural and social sciences involving radiation applications. Close contacts with professional associations and industries would provide information on attractive job opportunities. This would enhance the awareness among students and young professionals of the importance of radiation research, and contribute towards education, training, and the recruitment of future leaders in radiological protection. Such efforts could take advantage of the above-mentioned ICRP Mentorship Programme, which offers young undergraduates, graduates, and professionals to join current ICRP Task Groups where they can engage in scientific discussions with internationally recognised experts in the field of radiation sciences.

### Using plain language when interacting with the public and decision makers about radiological protection

The aim is to support people in making their own informed choices, increase the understanding of radiation risks and their uncertainties, and eventually contribute towards an objective view on the risks and benefits, for example, associated with technologies that use ionising radiation. Such efforts may benefit from resources provided by ICRP, for example, through the previously mentioned ICRPædia platform.

### Fostering general awareness of radiation and radiological protection through education and training of information multipliers

Educational/information multipliers include teachers, nurses, general practitioners, and other professionals. This could help members of society develop an unbiased view on, for example, how to best use ionising radiation, protect against existing exposures, and balance the risks and benefits against future societal needs and developments.

## Conclusions

All the issues mentioned above go hand in hand and require investment in training, education, research, and infrastructure in all aspects of radiation science related to radiation protection. Thus, all institutions, organisations, and initiatives active in the field of radiation research and radiological protection are encouraged to collaborate and identify synergies across disciplines and goals, establishing self-sustainable structures and making best use of the resources available to strengthen radiological protection.
